# Experiences of oldest-old caregivers whose partner is approaching end-of-life: A mixed-method systematic review and narrative synthesis

**DOI:** 10.1371/journal.pone.0232401

**Published:** 2020-06-09

**Authors:** Tessa Morgan, Aamena Bharmal, Robbie Duschinsky, Stephen Barclay

**Affiliations:** 1 Department of Public Health and Primary Care, University of Cambridge, Cambridge, England, United Kingdom; 2 Cambridge University Hospital NHS Foundation Trust, Cambridge, England, United Kingdom; University of Adelaide, AUSTRALIA

## Abstract

Population ageing has rapidly increased the number of people requiring end-of-life care across the globe. Governments have responded by promoting end-of-life in the community. Partly as a consequence, older spouses are frequently providing for their partner’s end-of-life care at home, despite potentially facing their own health issues. While there is an emerging literature on young-old caregivers, less is known about spouse carers over 75 who are likely to face specific challenges associated with their advanced age and relationship status. The aim of this review, therefore, is to identify and synthesise the literature concerning the experiences of caregiver’s aged 75 and over whose partner is approaching end-of-life. We conducted a mixed-method systematic review and narrative synthesis of the empirical literature published between 1985 and May 2019, identified from six databases: Medline, PsychINFO, Cumulative Index to Nursing and Allied Health Literature, Embase, Sociological Abstracts and Social Service Abstracts. Hand searching and reference checking were also conducted. Gough’s Weight of Evidence and Morgan’s Feminist Quality Appraisal tool used to determine the quality of papers. From the initial 7819 titles, 10 qualitative studies and 9 quantitative studies were included. We identified three themes: 1) “Embodied impact of care” whereby caring was found to negatively impact carers physical and psychological health, with adverse effects continuing into bereavement; 2) “Caregiving spouse’s conceptualisation of their role” in which caregiver’s navigated their self and marriage identities in relation to their partner’s condition and expectations about gender and place; 3) “Learning to care” which involved learning new skills and ways of coping to remain able to provide care. We identified a recent up-surge in published papers about very old spousal caregivers, which now comprise a small, medium-quality evidence base. This review outlines a range of potential lines of inquiry for future research including further clarification of the impact of caregiving on the likelihood of mortality, the incidence of men and women providing end-of-life care amongst this age group, and the role of anticipatory grief in shaping their perceptions of their relationship and their own longevity.

## Introduction

Rapidly ageing populations across the world present significant challenges to traditional health and social care models [1, 2], in no small part because people have more protracted end-of-life phases [3]. There has been a rapid rise of chronic diseases, including cardiovascular diseases, cancers, and respiratory disease, which continue to be the leading causes of death internationally [4]. Despite the fact that the majority of deaths internationally now occur amongst the over-65s, there remains relatively little policy concerning their needs and care preferences towards end-of-life [5]. This paucity of policy and research is starker still when considering the oldest-old [6–8], who are now the fastest growing age-group in developed countries [9].

Many governments are advocating the importance of providing end-of-life care in the community [10, 11]. This policy directive is informed by resource-limitations across health care sectors as well as more apparently empowerment-focused agendas of ‘personalized care’ and ‘ageing in place’ [5, 12]. Scholars working with family caregivers have voiced concerns that the ‘care in the community’ approach relies on family members to take on intense, often 24/7 care for indefinite periods of time [13–15] with input from professionals only late in the dying trajectory [16] if ever [17]. Alongside this care they are expected to manage their own feelings about the impending death of their relation [18, 19].

While there is some evidence that end-of-life caregiving can bring family members closer and catalyze caregiver’s personal growth [20, 21] other research suggests that family caregiver’s feel emotionally and physically unprepared for caring for their dying relative [22] and struggle with the financial consequences [23]. Marked associations have been identified linking caregiving with increased rates of depression [24], physical ill health [25] and mortality [26]. Additional evidence suggests these costs are disproportionately borne by women, who are more likely to spend more time caring and be engaged in more intense care tasks [27, 28]. Consequently women family caregivers have been identified at increased risk of psychiatric morbidity whilst caring [29, 30].

There is now a growing recognition in policy and research that family caregivers are often older themselves living with complex and multiple long-term conditions [3, 31–33]. The emergent evidence base on older caregivers of older care recipients indicates they are an at-risk population. For example, they are more likely to experience feelings of ‘powerlessness’ [34], and have an increased likelihood of caregiver breakdown [35]. Research to date has predominantly examined the experience of ‘third’ age caregivers aged between 60–75 [11, 36–39] or else includes caregivers 65 and above without differentiating further [22] despite evidence that there are important variations between being 65 to 75 let alone 65 to 90+ [40].

Comparatively little is known about oldest-old caregivers who are over-75. This is particularly concerning given that people in this age-group have a higher incidence of falls, dementia and declining social networks [8, 41, 42]: all factors likely to shape caregiving needs and experiences. There are also indications of unique gendered patterns of caregiving in this age group with UK census data suggesting that married men are more likely to be providing 50 hours or more of care per week than married women [43, 44]. This suggests there may be a different distribution of physical and psychological impacts and indeed meanings of caregiving amongst the very old. Greenwood and Smith’s [45] systematic review of oldest-old caregiving identified 18 published studies, of which most focused on early-stage dementia caregiving. They did not conduct a formal quality assessment of these studies, so the quality of the evidence remains unclear: nor did they examine the experience of providing end-of-life care specifically.

In order to gain an in-depth understanding oldest-old end-of-life caregiving, we decided to look specifically at spousal care. Oldest-old spouse caregivers have been identified as a particularly at-risk group when compared with non-spousal caregivers as they tend to: 1) provide more care per week [46], 2) live with the person they are caring [19] 3) provide more care as they age [47] 4) care without the support of other secondary carers [48] or formal services [49, 50]. Empirical research also indicates that older spousal caregivers are at greater risk of lower self-esteem [51], physical burden [52] and social isolation than adult-child carers [53, 54]. These disproportionately adverse impacts may be linked to the normative demands associated with living as a couple [55, 56].

Aim: to undertake a systematic review and narrative synthesis of the qualitative and quantitative literature published since 1985 concerning the experiences of oldest-old carers whose partner is approaching end-of-life.

## Research design and methods

### Outlining the search

Spousal caregivers were partners (whether married or not) who were “in a close supportive role who share in the illness experience of the patient and who undertake vital care work and emotion management” [57]. Participants were required to be community-dwelling rather than living in an institution to align with our own as well as policy-makers interest in end-of-life care in the community. Though various parameters have been used in the literature [58], this review defined oldest-old (or the ‘fourth age’) as aged 75 and above, in acknowledgement of differences in life-expectancies across the world (74 in East Asia and 80 and above in Western Europe) [12]. Our approach mirrors that taken by Greenwood and Smith [45] who included papers where the mean age of caregivers was 75 and over. When caregiver’s age-ranges might have a mean of 75 or over, full-text papers were read for clarification.

There are difficulties with the prognostication of end-of-life amongst the oldest-old, given protracted trajectories and difficulty surrounding when “really sick becomes dying” [5, 59, 60]. For example, in the case of dementia, while the condition is considered terminal upon diagnosis, it is usually in the later stages of the illness that people exhibits signs and symptoms of their end-of-life such as eating and talking less and sleeping more, although these signs may not directly result in immanent death [61].

As such, a more holistic definition was used in this review: “end-of-life” refers to the chronologically indefinite part of life when patients and their caregivers are encountering the implications such as symptoms and practical support needs of an advanced chronic or progressive life-limiting illness [62, 63]. Consequently, studies focusing on dementia were only included if they reported on moderate to severe stages of the illness. Papers focusing on diagnosis, early-stage or mild/moderate dementia exclusively were excluded.

### Search strategy

**Fig 1:**
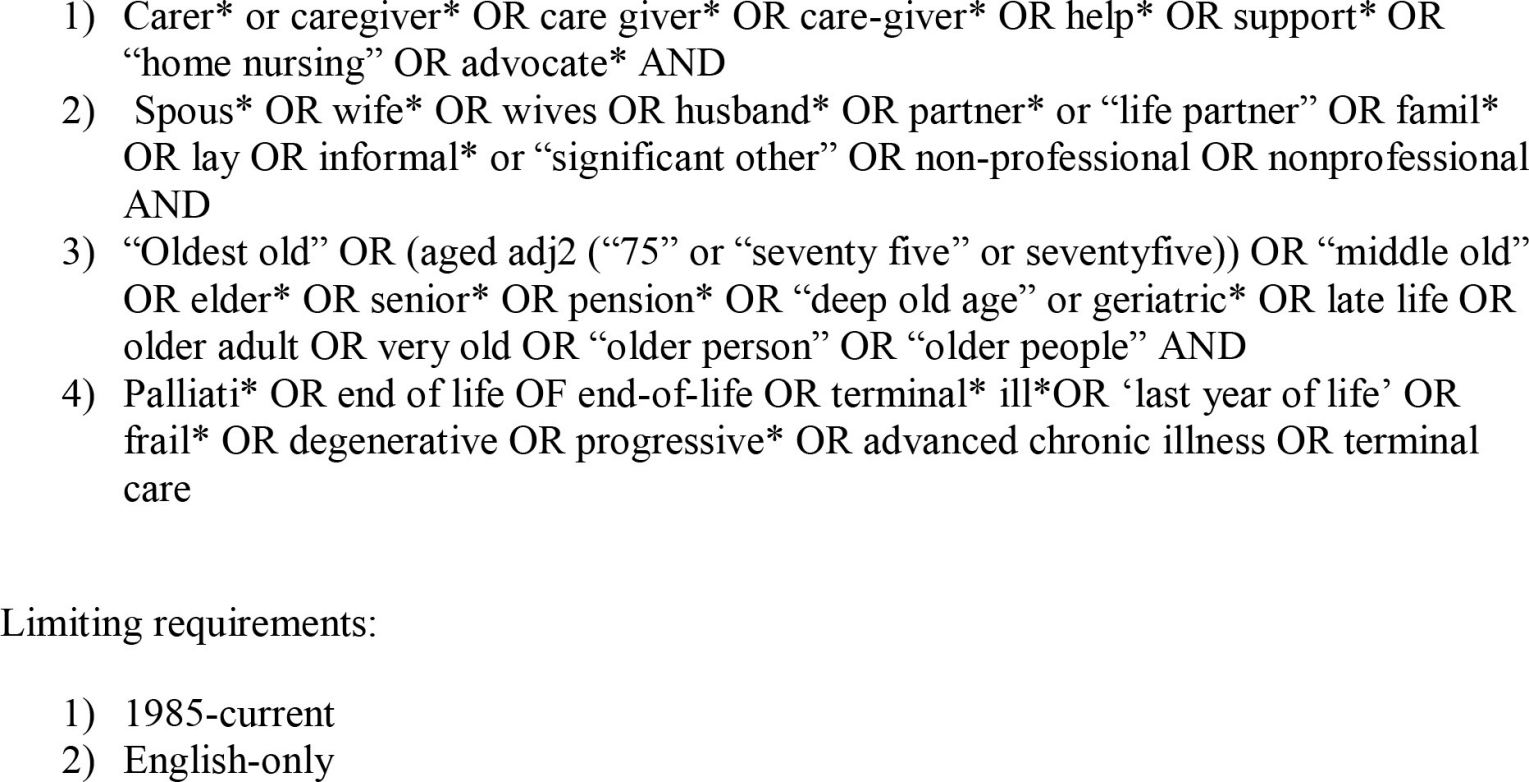
Search terms used in Medline.

The review protocol and search strategy was developed through discussion with the named authors and a professional Medical Librarian. The wider search process was guided by the PRISMA checklist (S1 Checklist)[64] and the research question was organised within the PICOS framework (participants, interventions, comparisons, outcomes, and study design). Search terms were initially developed in relation to the key search areas and subsequently refined through a pilot Medline search (Fig 1). Recognising the definitional complexity of the term “experience” [65], we followed a critical interpretative approach by not specifying predefined understandings of the concept in advance of the synthesis [66, 67].

The inclusion and exclusion criteria are outlined in Table 1. To maintain the focus on spouses as primary caregivers, included studies were restricted to home or retirement home settings, recognising the significant amount of independent living in the latter [68]. Hospitals, hospices and nursing homes were excluded as in these contexts health and social care professionals become the primary caregivers [69]. Included studies were peer-reviewed with substantively new empirical data. There was no restriction on methods and or country of origin. Papers were restricted to English language only as we had no resources for translation. Our search commenced in 1985 to align with ‘informal care’ and “family carer” entering bibliographic databases (Heaton, 1999) as a result of reduced public spending in many countries and a shift of responsibility for care of the elderly from the state to families and the voluntary sector [70]. A protocol has been registered with protocols.io: dx.doi.org/10.17504/protocols.io.bdm8i49w.

**Table 1:** Inclusion and exclusion criteria.

Inclusion	Exclusion
• Carer/ supporter/ helper of someone with life-limiting condition • Home, retirement village, Aged residential care facility • Carer 75 and above (mean of paper) • Search parameters: human, English-only, research, 1985- • Peer-reviewed, published empirical research • All research methods • No restriction on country of publication	• Perspective of person who is dying (primary focus) • Person being cared for does not have a broadly end-of-life condition (only a chronic illness e.g. arthritis) • Formal or paid health care professionals or volunteers. • Friends, adult-child, neighbours. • Hospital, hospice or inpatient unit at a retirement village • Mean of paper under 75 (unless case made for specific cultural relevance of oldest old in a particular sample) • Unpublished manuscripts, conference abstracts, posters and other empirical work not published in full Opinion pieces, guidelines, papers with no new empirical data • Grey literature • Interventions

### Conducting the search

In April 2018 TM searched six databases: Medline, PsychINFO, Cumulative Index to Nursing and Allied Health Literature, Embase, Sociological Abstracts and Social Service Abstracts. Between May-July 2018 and again in May 2019 TM independently screened titles to ensure the most up-to-date search. The title screening was done by one reviewer as is considered acceptable by Cochrane guidelines[71]. AB was a second reviewer through the abstract screening, full-text screening and analysis process. She was selected because she was not a content expert and thus did not have pre-formed opinions that can bias the assessment of the relevance and validity of articles. In line with advice from the medical librarian and Cochrane guidelines, AB searched a randomised selection of 10% of abstracts and a randomised selection of a third of full-text papers. TM and AB read and confirmed all of the included studies ahead of quality appraising all of these studies independently.

The transparency of the screening process was enhanced through the use of Rayyan a web application designed for collaborative citation screening and full-text selection [72]. Using Rayyan, TM and AB independently documented their inclusion or exclusion decisions by attaching a justificatory label to each paper (for example a frequent label was ‘excluded because of wrong age-range’). TM and AB subsequently conferred findings to ensure the consistency of screened studies. On the few occasions disagreements arose, the team (also involving two senior researchers) transparently resolved them by reference to reasoning recorded in Rayyan. Reference searching of included papers, citation searching using Google scholar, and reference chaining were then undertaken by TM to support robustness of the review.

### Data analysis

Included studies were analysed using a narrative synthesis approach in order to coherently and systematically integrate findings from studies using heterogeneous methodologies found in the included qualitative and quantitative studies [73]. This approach is suited to nascent fields as it provides a structured way to generate a ‘trustworthy story’ about the evidence base where little is currently known [73]. As oldest-old carers are an under-researched group this method was considered the most appropriate.

This narrative synthesis intertwined three main elements of Popay and colleagues’ approach [73]: developing a preliminary synthesis, exploring relationships within the data and assessing the robustness of the synthesis. Our adapted version of the narrative synthesis approach is presented in a supplementary table (S1 Table). Notable adjustments include conducting a critical appraisal of the data before the production of themes to ensure that the themes were not heavily weighted towards low quality studies or towards unique studies that had more than one paper included in the review.

TM and AB weighted the quality of each paper separately using Gough’s “Weight of Evidence”, a widely used tool suitable for qualitative and quantitative studies. This process involved rating studies ‘high’, ‘medium’ or ‘low’ in relation to the three categories A) generic quality of each studies, B) their specific appropriateness to the review, and C) their utility [74]. The ‘quality’ of each study was awarded on the basis of the average of the individual scores (if two highs and a medium study was marked high) (see Supplementary S2 Table) and is represented under the category ‘D’. TM and AB then compared their independently ascribed weightings of each study. Through this process we did not need to adjust the overall quality score (‘D’ rating) of any study indicating consistency in the quality appraisal between TM and AB.

Informed by evidence that end-of-life caregiving is a heavily gendered process [29, 43, 75], TM subsequently conducted a feminist quality appraisal of the evidence to determine how issues of power, gender and inequity (including those pertaining to intersecting identities of race, class and age) were handled in the aims, study design, data collection and analysis, discussion and recommendations for change section of each included study [76]. The studies were scored and the quality attributed through the same process outlined for Gough’s tool.

We conducted a thematic analysis that focused on the “main, recurrent and/or most important (based on the review question) themes and/or concepts across multiple studies” [73]. Through this process we identified three overarching themes. These are presented below. To further protect against bias, a modified version of the vote-counting process was used to determine whether each theme was supported, negated or irrelevant to each included study in turn. Relevant insights from this process, particularly the cases of conflicting findings, were subsequently incorporated into the synthesis. Themes were frequently discussed between TM, AB, SB and RD to aid transparency and reliably of their production.

## Results

**Fig 2:**
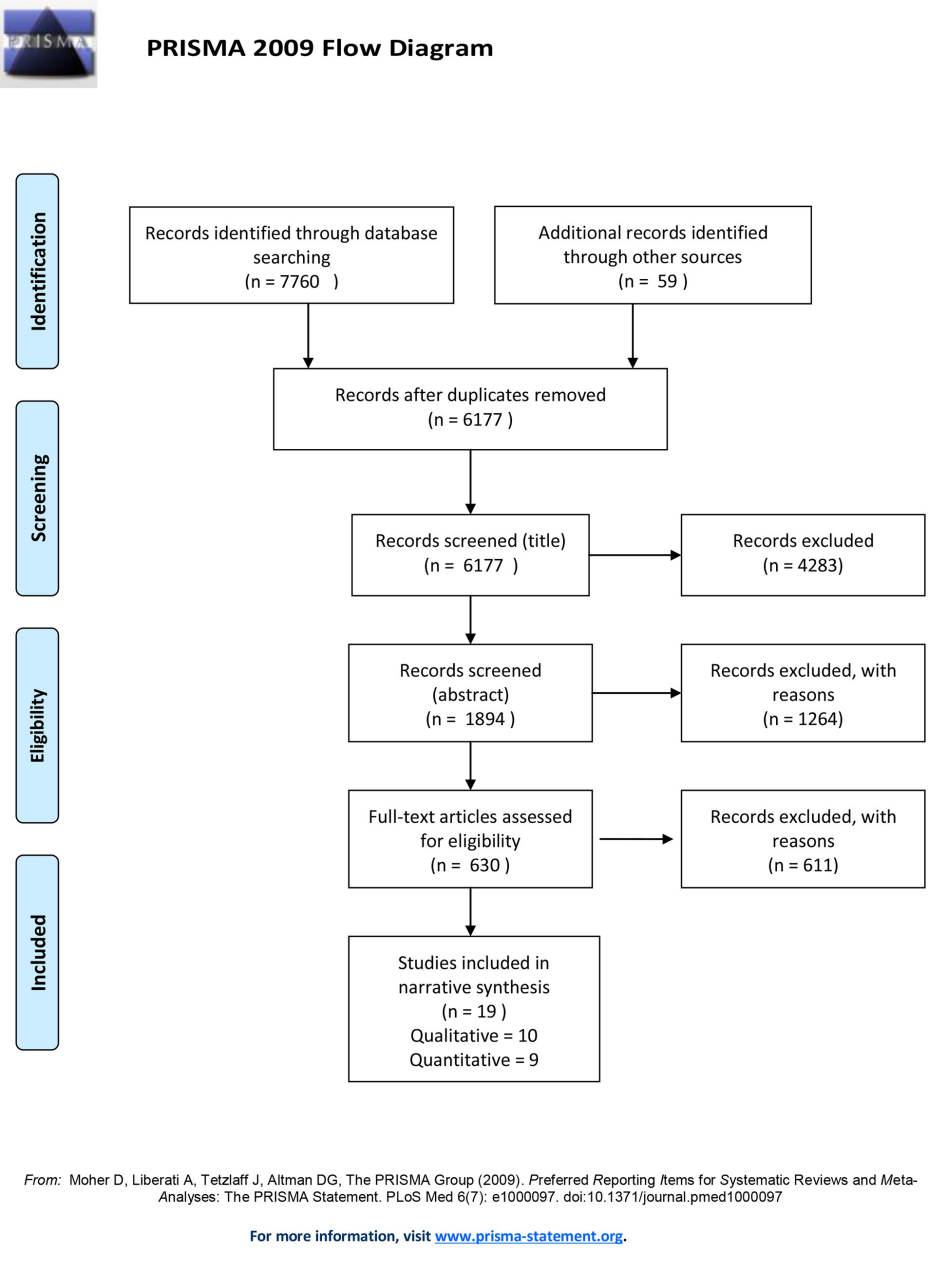
PRISMA flow diagram.

Search results are summarised in the adapted Preferred Reporting Items for Systematic Reviews and Meta-analyses (PRISMA) flowchart (Fig 2 and S1 Checklist) [64] and the characteristics are displayed in supplementary S2 Table. A total of 19 papers were included from 16 unique studies, of which 10 used qualitative methods and nine quantitative methods. It was striking that 10 of the papers included were published in the last three years (between 2016–2019) and none were published before 1993 [77].

Based on Gough’s “Weight of Evidence” all but one of the included papers were of at least medium quality. Four papers drew from large longitudinal observational cohort studies covering two, three, seven and eight time points [77–80] and one study [81] included a retrospective longitudinal analysis of general practice records (median time 3 years). Three qualitative studies conducted serial interviews [82, 83] including an ethnography lasting 13 months [84]. The feminist quality of included papers was low despite the frequent focus on the differences between genders: only six studies engaged with the gendered construction of care [83, 85–87] and only two did so to a high standard [88, 89].

Of the nine papers providing mean ages of caregivers, the collated mean age was 76.9 years [77, 79–81, 83–85, 90, 91] (note this only includes dementia carers in Dassel studies). Of the five studies reporting age ranges, all but one included at least one caregiver aged over 90 [82, 86, 88, 89, 92]. Studies reported from exclusively western contexts and from predominantly white and heterosexual populations. Thirteen studies reported, at least initially, on current caregivers, three studies focused on bereaved carers and three studies combined both groups of caregivers.

Most studies identified their caregiver-participants via their partners’ condition with only four studies requiring participants to self-identify as carers/ caregivers [79, 80, 83, 90]. Only four studies provided a definition of caregiving, with three definitions focused on supporting another person in their activities of daily living [77, 83, 91] and one study requiring caregivers to be registered for the government-funded carers’ allowance scheme [90]. One study that identified caregivers by virtue of co-habitation with a person at their end-of-life reported that only 6.9% of this sample had been formally identified as a caregiver by their General Practitioner (GP) (a community based doctor who treats patients with minor or chronic illnesses) [81].

Participants were caring for spouses with a range of end-of-life conditions with six studies focusing exclusively on care-recipients with end-stage dementia [82, 85–88, 91] six on advanced frailty [77, 78, 83, 90, 93, 94], and the rest focusing on care-recipients who had died. Both the age of the care recipient and the length of care were inconsistently reported with eight and 10 studies respectively not providing this information.

### Narrative synthesis of content

This section presents a narrative synthesis of the overarching themes identified across the included studies. The three themes were the embodied impact of care, caregiving spouse’s conceptualisation of their role, and learning to care.

#### Embodied impact of care

Studies highlighted the ‘double jeopardy’ [84] associated with caring for a spouse whilst managing one’s own poor health. Studies reported caregivers with multiple chronic comorbidities, frailty, respiratory problems and, in one study, cancer [79–81, 84, 85, 90, 92]. In three quantitative studies, caregiving increased the risk of frailty [78, 79], with one study indicating that caregivers were six times more likely to be frail than non-caring peers when controlling for other factors [94]. Two studies reported carers to have been admitted to hospital with their own poor physical health, which they had neglected in order to continue caring for their partner [84, 92].

Caring was linked with high levels of emotional stress [82, 86, 88] and psychological strain [80, 81, 85, 90]. Qualitative studies depicted end-of-life care as an exhausting 24/7 role, and both qualitative and quantitative studies highlighted the socially isolating nature of care [80, 84, 86, 92, 94, 95]. Having cared for one’s spouse at end-of-life continued to negatively impact the health of caregivers years after caregiving had ceased following bereavement. Bereaved older caregivers had increased prescriptions for antidepressant and antianxiety medication and more GP consultations than non-carers [81] and caregivers of those with dementia were found to be particularly at risk of their own cognitive decline [80]. Whereas one longitudinal observational cohort study concluded that having cared for a partner increased the risk of mortality [80], another longitudinal review of GP records did not find a significant correlation [81]. Notably, however, caregivers who had severe health problems, such as dementia or depression, were excluded from studies either explicitly [79, 80, 90] or implicitly due to requirements around capacity to consent, raising question about whether health impacts of providing care may currently be under-reported. Conversely, some qualitative studies conclude that spouses viewed their caregiving as ‘life-sustaining’ and the reason for their own longevity [82, 83].

#### Caregiver’s conceptualisations of their role

Caregiving was seen as a new chapter of the spousal relationship, which was undertaken out of a combination of their love for their partner and/or an obligation associated with their marital vows [82–84, 86, 87, 90–92]. Caregiving spouses strived to maintain familiar aspects of the couple’s daily interactions and routines predating the on-set of their partner’s illness. Central to this was caring for their partner at home [82, 83, 91, 92], which they often did with little formal or informal support [77, 82, 85, 86, 88]. Nevertheless, as their partner’s approached end-of-life, caregiving spouse’s found it difficult to maintain aspects of their self and marriage identities [82, 86, 87, 91, 92]. On a practical level, caregivers had to perform more care and modify their house and social life in unfamiliar ways [82, 91]. They also often had to grapple with the losses associated with declining communication and sexual intimacy with their spouse [79, 80, 83, 86–88, 91, 95]. One study explicitly identified such loss of intimacy as a trigger for anticipatory grief [86].

For dementia caregivers this also involved taking on new roles such as protector of their spouse’s dignity and personhood [82, 83, 87, 91]. Caregiving spouses also struggled when they felt they could not share negative aspects of the caregiving with their partner [86, 90] including their fears and anticipatory grief associated with their partner’s imminent death [82, 83, 86]. Caring could be particularly difficult where relationships had been strained prior to illness [87, 90, 91].

Caregiving appears to be conceptualised and experienced differently across genders. Given cultural discourses around women’s innate caring nature [87, 89] and their history of having provided care to other family members [91], wives largely took it for granted that they would care for their husbands at end-of-life. Studies reported that women were more willing to sacrifice their own health and social needs to their partners [83, 84, 89, 91]. Studies indicate that husbands tended to initially struggle with the repetitive and thankless nature of caring and household tasks but were able to subsequently incorporate their care into their masculine identities, by reframing nurturing within their pre-existing management skills [85, 86, 88].

#### Learning to care

Studies highlighted that spouses had to learn to provide end-of-life care for their spouse. They had to become experts on their partner’s condition and coordinators for their care. This often involved navigating multiple care systems and dealing with a variety of health care professionals inside and outside of the home [84, 86, 88, 90–92]. Many older caregivers tended to provide most of their partner’s care themselves, especially when their partner had a non-malignant condition, which meant they received less specialised support [80, 92]. Sampson [81], however, found that GP surgeries offered similar levels of support to caregivers, regardless of their partners’ condition, provided they were both registered at the same practice.

Caregivers tended to take on new forms of hands-on care. They had to learn to provide for their partner’s personal care [83–85, 92] or had to organise for outside services to do so, a strategy more commonly adopted by men [77, 86, 88]. Caregivers frequently had to make practical changes to their homes including bells and call systems [91, 92].

Husbands and wives often reported taking on new aspects of household management and maintenance that their spouse had previously performed. Men reported learning new tasks such as food preparation, cleaning and organising social activities [82, 83, 86, 88], women reported becoming independent decision makers and financial managers [84, 91].

Oldest-old caregivers also had to learn coping strategies to keep caring. Studies acknowledged a range of emotional coping strategies caregivers utilised such as reminding oneself of the purpose of caring when frustrated [82, 86, 88, 91], drawing on humour [92] and instrumental support to overcome particularly difficult tasks [85, 88, 92]. Caregivers were forced to sharpen their decision-making skills and crisis-management capacity [84, 92].

Part of coping also entailed the caregiver identifying their limits. Three studies found that caregivers were more likely to use formal carers for personal care and make use of respite services when their partners were closer to the end-of-life [86, 88, 92]. Nevertheless, one study found co-resident caregivers used less formal services than those not living with the partner irrespective of the patient’s condition [77]. Dementia caregivers often discussed their plans for moving their spouse into residential care. Studies reported some caregivers who did so when they could no longer cope though these studies also stressed that these caregivers made sure to remain involved in their spouse’s care [78, 83, 86, 88, 91].

## Discussion and implications for research, policy and practice

This is the first literature review to systematically collate, narratively synthesise and quality appraise the extant literature on oldest-old spouses providing end-of-life care. We identified an upsurge of research published in the last three years, attesting to the growing interest in the academic community of putting oldest-old spousal caregivers on the policy agenda [78, 80]. Indeed, the fact no study was published before 1993 may also indicate that end-of-life caring amongst the very old is a particular product of present-day conditions where people reach their end-of-life in advanced age and are expected to be cared for in the community.

The evidence presented in this review is of medium quality on Gough’s Weight of Evidence, which is perhaps higher than expected for a nascent sub-field. Researchers have seemingly heeded calls in the caregiving literature for more longitudinal research that captures the important temporal aspects of caregiving over the development of an illness and life-cycle [32, 96]. A further explanation is that a notable proportion of these studies are secondary analyses of high-quality ageing cohort studies. However, because these oldest-old caregivers were an unanticipated finding of these studies definitions of ‘caring’ and ‘carer’/ ‘caregiver’ may not have been as sufficiently outlined to make them high quality studies in light of this review’s focus.

On the other hand, the feminist quality of these studies is low and leaves many questions unresolved. This potentially reflects a similar lack of gendered analysis in end-of-life care research which is where a high proportion of these studies originated [29]. As such, the incidence of men or women providing end-of-life care in this age group requires further attention; particularly in settings other than the UK. This review has been unable to corroborate or deny earlier findings that men in this very old age group provide more care [43]. The physical and psychological impact of caregiving as analysed by gender also requires further attention. Nonetheless, studies described a degree of improvisation in the way these caregivers ‘do’ gender, challenging assumptions that they necessarily follow ‘traditional’ gendered scripts because they are members of the stoic post-World War II generation [97]. These findings suggest we have far more to learn about how this group’s advanced age, gender and other aspects of identity characteristics intersect to shape their caregiving experience [89].

In line with previous studies, we found that spousal caregivers 75 and over provided a wide-range of care for their partner including administration and advocating, emotional support and hands-on care tasks [14, 16, 19]. This review fits with current policy indicating that the high level of care provided by older spousal caregivers continues into the last stages of life [47]. Echoing previously reported findings, included qualitative studies highlighted the centrality of spouses developing a range of coping mechanisms to facilitate their caring with obstacles to care seemingly framed as ‘challenges’ rather than ‘threats’ [98, 99]. From this perspective, spouse’s active decision to move their partner to a care home could be viewed as a coping tactic employed to sustain rather than stop their caregiving [83, 91]. More research is required around the impact of these transitions on the continuity of care provided by spouses and is particularly relevant for this age group whose own competing health issues may increase the likelihood of them either separately or jointly having to move into a care home [100, 101]. It was notable that there was little mention of pain and symptom management, which is commonly the focus of end-of-life care [57]. This possibly reflects included studies focus on long-term conditions such as severe dementia and advanced frailty where mood and comfort control are the most relevant to the caregiving experience [102]. Overall, recognising the expertise caregivers gain over the course of their partners illness would serve as a valuable resource for health and social care professionals involved in their spouse’s care and align with wider policy incentives of see caregivers as ‘co-workers’[103].

Evidence suggests that in most cases oldest-old spouses care out of a mixture of normative expectations to do so and out of love of their partner. Qualitative studies emphasised caregiver’s desire to sustain their self- and marriage-identities built up over their life course and favour their spouse label over their caregiver role [22, 35, 104]. In striving for a coherent form of self in spite of the caregiving responsibilities included studies suggest a tendency of very old caregiving spouses to privilege *biographical flow* whereby illness is incorporated into on-going life and identity over *biographical disruption* where illness disrupts and dominates one’s sense of self and everyday life [105, 106].

The concept biographical flow also helps to explain why the home and household chores featured so frequently in discussions about care. The home has been found in previous research to offer a familiar anchoring point against the ‘persistent liminality’ accompanying both advanced old age and the end-of-life period [107]. Moreover, spouse’s attempts to maintain familiar aspects of their everyday lives for as long as possible might explain some couple’s reluctance to utilise formal services despite struggling to provide care alone [17, 50, 88]. Another explanation for their unaided caring may be that their partner is not being offered services because they are lower priority given their age and non-malignant conditions [17]. More research is needed in this area, including the views of service providers [75] as well as from a gendered perspective given wider evidence that indicates men tend to receive more formal and informal support whilst caring than women [30, 108].

Where *biographical disruption* was reported, it appeared to be precipitated by actual or expected changes in communication and intimacy between partners rather than the biological or cognitive change of their partner [105, 109]. This indicates that health care providers should be particularly attuned to providing support and/or strategies to enable spouses to maintain their verbal and non-verbal communication. To do so health care professionals should ‘think couple’ when designing support strategies that include both members and facilitate opportunities where couples can be observed together, for example at joint GP visits or community groups for both spouses. In some cases, bereavement support might usefully be brought in earlier to help both spouses to manage anticipatory grief and help the caregiving spouse cultivate strategies for when their caring responsibilities cease [38]. Future research is also required to ascertain whether anticipatory grief is more pronounced in this group given their advanced age and the physical impact of caregiving may increase the likelihood they are also approaching their end-of-life whilst caring for their partner [8]. This could be a reason for very old caregiver’s emphasis on biographical flow as a form of ‘ontological security’ which prominent sociological scholar Anthony Giddens defines as the ‘stable mental state derived from a sense of continuity in regard to the events in one's life’[110].

The substantial physical and psychosocial impact of caregiving on spouse’s health reflects just how illness impacts the whole family, not just the person approaching their end-of-life [96]. This review also suggests in line with previous research that this is a group who are particularly at risk because of their age and pre-existing conditions [8]. Indeed, one observational Belgium study found caring was linked with increased rates of mortality [80], although, another UK retrospective GP-record study reported a non-significant increase [81]. Further, preferably multi-centred, research is required to clarify whether observed increases in mortality are context-specific, a result of different methodological choices or a more generalizable phenomenon. Researchers could usefully explore ways in which caring can be experienced positively and investigate the situations where it can be ‘life-sustaining’ to ensure that caregiving is not pathologized ipso facto [20].

Research is also needed to understand the extent to which very old caregivers themselves have end-of-life and/or terminal conditions. This review provides glimpses of such caregivers–for example those with dementia–however only because they were excluded from such studies. Given age is one of the biggest risk factors for developing dementia the phenomena of the person with dementia being the caregiver may be an urgent area of future research [41]. Including caregivers with mild/moderate dementia via methods such as process consent [111] would help to ascertain a fuller picture of the psychological and physical experience of caregiving. It may also help clarify the degree to which co-caring occurs between spouses [104]. By viewing caregivers as potential ‘co-patients’ with their own health and service needs researchers can contribute to growing recognition that *vulnerable dyads* need to supported before a crisis occurs and one or both are expectantly admitted to hospital, care home, or die [112, 113].

Finally, it is important to recognise that the insights presented above draw from the experiences of a relatively homogenous population. Like Greenwood and Smith [45], we found the academic literature largely reflects the experiences of white, heterosexual married couple in affluent countries. Experiences of those caring in their late 80 and 90s were similarly under-represented. Future research needs to specifically include the experience of diverse caregivers in a range of settings to ensure policies are culturally inclusive and appropriate [11, 14]. More sociologically and/or anthropologically informed research would help to provide a theoretical basis for unpacking such cultural specificities of caregiving [32]. Oldest-old non-spousal caregivers, including children, friends and neighbours, also need to studied, given that their caring is likely governed by different social mores than spouses [54], and included other challenges such as transportation if they do not live with the person they care for [51].

### Limitations

We recognise that using the mean age of 75 to determine the experiences of oldest-old is an imperfect measure shaped by the realities of inadequate reporting of age in studies and little exclusive focus of the very old. This pragmatic strategy meant we were able to isolate this age group somewhat although we recognise that some insights reported may very well be shaped by evidence from the young-old [45]. In addition, all of the papers identified during reference searching centred on people with severe dementia. In discussion with our medical librarian it was found that these papers were not captured by the database searches because they did not contain the end-of-life terminology [62]. This may reflect an academic and service provision reticence to address ‘dying from dementia’[114]. Inclusion of ‘severe’ or ‘end stage’ dementia would be useful additional search terms when conducting future systematic reviews of this age group. Nonetheless, we are confident that our multi-level search strategy effectively captured all the available evidence pertaining to our review question. A final limitation of this search is that one reviewer conducted the majority of title and abstract screening. This was primarily because this review was conducted as part of the first reviewer’s PhD. As outlined above a number of steps were taken to mitigate bias including consulting a medical librarian, drawing on guidance of Cochrane guidelines when deciding how much reviewing a second reviewer ought to do, consulting as a team at every step of the process and using the Rayyan application that enabled a clear audit trail. Finally independent screening and quality appraising of all included full-text papers by both reviewers also enhanced rigour.

## Conclusion

This is the first systematic review to synthesise and appraise the published literature concerning oldest-old spouses providing end-of-life care. The small, medium-quality evidence base attests to the range of physical, psychosocial and existential challenges facing oldest-old spouses that result from caring for their dying partner at home. More theoretically-informed research with a more diverse range of spousal caregivers is required to capture the variety of caregiving practices amongst the very old. Service providers and policy makers could usefully ‘think couple’ when designing strategies that support spouses to continue to care for their partners. Finally, researchers and service designers alike need to develop new ways of engaging with oldest-old spousal caregivers who are often at once ‘co-workers’ in their partner’s care and ‘co-patients’ with their own health issues.

## Supporting information

S1 Checklist PRISMA checklist. (DOC)

S1 Table Adapted narrative synthesis approach. (DOCX)

S2 Table Characteristics of included studies. (DOCX)
