# Supplementary material for: Experiences of oldest-old caregivers whose partner is approaching end-of-life: A mixed-method systematic review and narrative synthesis
Source: PLoS One. 2020 Jun 9;15(6):e0232401. doi: 10.1371/journal.pone.0232401 (PMC7282625; doi:10.1371/journal.pone.0232401)
Supplement: S1 Table — (DOCX) [file pone.0232401.s002.docx]

| **Analysis** | **Description of how it was followed** |
| --- | --- |
| Textual descriptions of studies | Annotated bibliography of all included studies created which included main findings and an initial reflection from lead analyst about content and quality of each study. |
| Tabulation as means of transforming data into common rubric | Table created to display characteristics of quantitative and qualitative studies, which enabled initial mapping of the characteristics of studies. This helped to identify the relative homogeneity of research participants, which is relevant to later stages of analysis. |
| Assessing robustness of studies using two quality appraisal tools. | Decision made to assess the quality of studies before the synthesis was conducted to ensure that the evidence underpinning the synthesis was not biased on low quality studies.  Using Gough’s weight of evidence enable us to determine the general quality the studies methodologies as well as the extent to which they contributed to answering the aim of the review.  The Feminist quality appraisal tool enabled a consideration of how power, inequity and gender were considered at each stage of the process (for example, who is included in each study, what theoretical framework are utilised within studies). This approached was used as it reflected our hypothesis of the ‘theory of change’ insofar as we expected that gender would shape the experience of caregiving. |
| Groupings and clusterings | Three distinct types of studies were identified:  1) Caring retrospective vs. prospective  2) Main focus was physical/psychological impact vs. everyday practice of caregiving  3) Dementia vs. non-dementia studies. |
| Translating data: content analysis | Summary descriptions of qualitative and quantitative studies were produced. These were further refined and combine to characteristics of study methodologies and qualities of the carers and care recipients included in studies. |
| Translating data: thematic analysis | A thematic analysis was conducted with above groupings in mind, to consider the “main, recurrent and/or most important (based on the review question) themes and/or concepts across multiple studies”(Popay et al. 2006, p. 18).  For example, themes included:   1. Physical impact of caregiving 2. Maintaining normality 3. Findings way to cope   Overarching latent themes were then identified by considering the underlying concepts connecting themes. These included:  1)Embodying care  2) Conceptualising care  3)Learning to care |
| Vote-counting (with textual data) | This approach was adjusted so it could be applied to textual as opposed to statistical data. Where statements were made about the nature of caregiving (for example, that it increases one’s risk of mortality or that it was informed by a desire to maintain normality) each study relevant to the statement was inspected to see if it affirmed or contravened the statement. Studies supporting the claim were inserted as references in-text. Where statements were in dispute this was made explicit.  This approach safe-guarded against focusing too much on one or two studies. |
| Sub-group analysis | Gendered sub-group analysis was undertaken – aided by the feminist appraisal tool which sensitised us initially to any gendered discrepancies reported in each study.  In addition, each theme was considered in relation to the three groups identified at the ‘grouping and clustering’ stage. |
| External validation | A version of this manuscript was delivered at two conferences with practitioners to determine whether the key narratives presented were credible and recognisable from the perspective of health care professionals and other academics with an interest in this population group. This helped to solidify that what is presented in this publication is not only a “trustworthy” but also a relevant story. The wider conceptualisation of this research project and its research questions have also been shaped by two formal Patient and Public Involvement (PPI) groups which are focused around experiences of palliative care and older people’s health respectively. |
