# Supplementary material for: Experiences of oldest-old caregivers whose partner is approaching end-of-life: A mixed-method systematic review and narrative synthesis
Source: PLoS One. 2020 Jun 9;15(6):e0232401. doi: 10.1371/journal.pone.0232401 (PMC7282625; doi:10.1371/journal.pone.0232401)
Supplement: S2 Table — (DOCX) [file pone.0232401.s003.docx]

| Study details | Aims and objectives | Study design | Caregiving spouses’ characteristics | Care-receiving spouses characteristics | Context of care | Key findings | Quality |
| --- | --- | --- | --- | --- | --- | --- | --- |
| Orit Shavit, Aaron Ben-Ze’ev, Israel Doron (2019) Love between couples living with Alzheimer’s disease: narratives of spouse care-givers *Ageing & Society* 39: 488-517. | To describe and analyse the meaning of love in relationships between couples living with Alzheimer’s disease (AD). | Qualitative narrative approach. Semi-guided interviews. Thematic analysis (focused on case-based analysis). | 16 spousal carers.  Mean age carer= 75.25.  Gender = 8 women, 8 men.  Ethnicity= A range of origins listed including 5 Israeli, 1 Syrian, 1 Moroccan, 1 Yemeni. Note that Arabs were excluded. | All had moderate to severe AD.  Mean age = 77.8.  Mean Duration of caregiving = 4.9 years.  Mean length of marriage 51.81 years. | Israel.  Home-based care. | Love takes on many forms in face of AD (can enhance, can diminish, may not have been there at all).  Intimacy gained different meaning as a result of disease. Not being able to communicate resulted in a continuum of ‘we’ and ‘I’ narratives from carers about their relationship. | A= Medium  B= High  C= High  D= High  Feminist appraisal = Medium. Main author reflexively discusses her positionality. The social construction of love and being a good wife explored. Potential ways of alleviating issues with intimacy suggested. |
| Florence Potier, Jean-Marie Degryse, Benoit Bihin, Florence Debacq-Chainiaux, Chantal Charlet-Renard, Henri Martens, Marie de Saint-Hubert (2018) Health and frailty among older spousal caregivers: an observational cohort study in Belgium *BMC Geriatrics*, 18: 291-300. | Longitudinally assess the health of older spousal caregivers considering frailty, nutrition, cognition, physical performance and mood disorders. | Longitudinal, observational cohort study. Range of clinical instruments such as Frailty Phenotype and Mini Nutritional Assessment and Zarit Burden Index.  Confidence intervals between baseline and second survey used to analyse change over time. | 82 community-dwelling spouses.  Mean age at baseline =  80 (range = 77-85).  Gender = 54% women, 46% men.  Ethnicity not reported. | 83% of care recipients had cognitive impairment, 68% with behavioural disorders and most with moderate frailty on Katz index.  27/78 dead at 16 month follow-up stage.  Mean age = 81 years. | Wallonia, Belgium.  Home-care at baseline. Only 51/78 at home at 16 month follow-up.  Duration of caregiving not reported. | 1/3 caregivers frailty status worsened. Of particular concern was unexpected weight-loss and lower activity.  Nutrition, cognitive status, reported level of burden and mood assessments all stayed stable. | A= Low  B= Medium  C= Medium  D= Medium  Feminist appraisal: Low.  Indicates no differences between genders in terms of nutrition and notes of caregivers no longer giving home care 21/27 female. No gendered analyses provided. |
| F Potier, J-M Degryse, G. Aubouy, S Henrard, B Bihin, F. Debacq-Chainiaux, H. Martens, M. de Saint-Hubert (2018) Spousal caregiving is associated with an increased risk of frailty: a case-control study *The Journal of Frailty & Aging* 7(3): 170-175. | Explore if older spousal caregivers are at greater risk for frailty compared to older people without a load of care. | Cross-sectional analysis of baseline data from cohort study.  Multivariate logistic regression analysis.  Variables compared between caregivers and controls using Mac Nemar test for categorical variables and Wicoxon signed rank test for continuous measures. | 79 older spousal carers compared to control group of non-carers.  Mean age carers 79 (range 76-84)  Mean age control group 78 (75-82).  Gender = 53.2% women for both carers and control group.  Ethnicity not reported. | 82% had cognitive impairment, 68% with behavioural disorders.  27/78 dead at 16 month follow-up stage (insight drawn from companion study).  Care-receivers age (M= 81.4 SD 5.2). | Wallonia, Belgium.  Home-based care.  Duration of caregiving not reported. | Controlling for age, gender and comorbidities, caregiving is associated with a risk of frailty (6x that of non-carers), the consumption of antidepressants, shorter nights sleep and more difficulties maintaining social network.  Caregivers’ frailty not associated with cognitive or functional status of care-receiver, although it was associated with nurse at home. | A= Medium  B= Medium  C= High  D= Medium  Feminist appraisal = Relatively equal proportion female and male. Nutrition not found to vary despite expectation. No gendered analyses provided. |
| Florence Potier, Jean-Marie Degryse, Severine Henrard, Genevieve Aubouy, Marie de Saint-Hubert (2018) A high sense of coherence protects from the burden of caregiving in older spousal caregivers *Archives in Gertontology and Geriatrics* 75: 76-82. | Study the links between sense of coherence (SOC), burden, depression and positive affects among caregivers of frail older patients. | Cross-sectional analysis of baseline data from cohort study.  Multivariate logistic regression analysis. Caregiver’s characteristics were analyzed by burden severity and SOC level. | 79 older spousal carers.  Mean age = 79.4 (SD 5.3)  Gender = 53.2% women, 46.8% men.  Ethnicity not reported. | 82% had cognitive impairment, 68% with behavioural disorders, and moderate frailty.  27/78 dead at 16 month follow-up stage (insight drawn from companion study).  Mean age = 81.6 (SD 5.3). | Wallonia, Belgium.  Home-based care.  Median duration of caregiving = 3 years. | Older caregiver age and high SOC associated with lower caregiver burden.  Higher burden among carers of care recipients who had more ADL dependence.  No difference socio-economic status, gender and clinical measures, such as the comorbidities, cognition, physical performance, frailty and malnutrition. | A= Medium  B= Medium  C= High  D= Medium  Feminist appraisal = Low. Mention that gender not found as difference. No deeper explanation provided. |
| Carol Thomas, Mary Turner, Sheila Payne, Christine Milligan, Sarah Brearsley, David Seamark, Xu Wang, Susan Blake (2018) Family carers’ experiences of coping with the deaths of adults in home settings: A narrative analysis of carers’ relevant background worries *Palliative Medicine* 32(5): 950-959. | To illustrate the relevance of ‘relevant background worries’ in family carers’ accounts of caring at home for a dying adult. | Qualitative cross-sectional observational study. In-depth, semi-structured interviews. Narrative analysis of a subset of transcripts from wider study (N=59). | 30 family carers.  Median age:  70-79  Gender= 23 women, 7 men.  Ethnicity = 28 White-British, 1 White-European, 1 White-North American. | Older dying adult with malignant and/or non-malignant condition.  Median age = 80-89.  Gender = 21 Males, 9 Females. | England, United Kingdom.  Home-based care.  Median duration of caregiving = 11 months. Range 2 weeks to 11 years. | The importance of discussing ‘relevant background’ worries such as discussing death, losing friends during and after caring and fears associated with partner forgetting who you are. | A= High  B= High  C= High  D= High  Feminist= Low. No specific gendered analysis offered. |
| Kara B. Dassel, Dawn C. Carr, and Peter Vitaliano (2017) Does Caring for a Spouse With Dementia Accelerate Cognitive Decline? Findings From the Health and Retirement Study, *The Gerontologist*, 57 (2), 319-328. | Examine whether dementia compared to non-dementia spousal caregiving is related to cognitive health. | Quantitative longitudinal data from 8 biannual waves of the telephone Health and Retirement Survey (1996-2010).  Bivariate analyses, ordinary least square regression applied to 2 waves before and 2 waves after death of care recipient. | Total sample 1255 surviving spouses, 192 cared for spouse with dementia.  Mean age of dementia carers = 76.417 (SD 8.9). Mean age of non-dementia carers 72.38 (SD 9.67).  Gender = 74% women, 26% men.  Ethnicity = 80% non-hispanic white. | All had died within the period of the study.  With dementia = 192.  Without dementia = 1063.  Age not reported. | United States.  Home and rest home based.  Duration of caregiving not reported. | Controlling for baseline health and contextual factors (e.g., frailty status, age, education), dementia caregivers had significantly greater cognitive decline (*p* < .01) compared to non-dementia caregivers.  Dementia caregivers cognition continued to decline after caregiving tasks has ceased. Dementia caregiving may trigger an underlying neuro- pathological disease process. | A=High  B=Med  C=High  D=High  Feminist analysis – Low, no discussion of gender. |
| Lisa Ann Williams, Lynne S Giddings, Gary Bellamy and Merryn Gott (2016) ‘Because it’s the wife who has to look after the man’: A descriptive qualitative study of older women and the intersection of gender and the provision of family caregiving at the end of life, *Palliative Medicine*, 31(3), 221-230. | To explore how gender norms constructed older women’s views about the appropriate roles of women and men in providing palliative and end-of-life care for family members. | Qualitative focus groups using vignettes around advance care planning. Thematic analysis. | 39 older adults.  Age range 50-99. Median between 70-79.  Gender = 36 women, 3 men.  Ethnicity of participants: NZ European/ Māori = 1; Niuean = 1; Irish = 1; Australian = 1; NZ European = 31. | Not specific about those who participants had cared-for other than stating that they were end-of-life.  Age not reported. | Auckland, New Zealand.  Range of care locations.  Duration of caregiving not reported. | Caregiving was heavily tied to normative ideas about gender.  It was taken for granted by female participants that women should provide end-of-life care.  Women were viewed as naturally adept to care whereas men were not.  Recognition that in their own lives,  adhering to norms doesn’t equate to being happy with them. | A=High  B=High  C=Medium  D= High  Feminist analysis- High, considering societal constructions of femininity and masculinity and how this plays out in everyday life, for example, through the use of humour. |
| Kara B. Dassel and Dawn C. Carr, (2016) Does Dementia Caregiving Accelerate Frailty? Findings From the Health and Retirement Study, *The Gerontologist*, 56 (3), 444-450. | To examine the relationship between care-recipient and whether, compared with the wave prior to death of the care-recipient, spousal caregivers were frailer: 1) in the wave the death was reported and 2) 2 years after the death was reported. | Quantitative longitudinal data from 7 biannual waves of the telephone Health and Retirement Survey (1998-2010).  Bivariate analyses and logistic regression models applied to 1 wave before and 2 waves after death of care recipient. | 1246 surviving spouses.  Mean age of dementia carers = 75.97 (SD 9.152). Mean age of non-dementia carers = 71.87 (SD 9.863).  Gender = 73% women, 27% men.  Ethnicity = 78% Non-hispanic White, 12% non-hispanic black, 7.5% Hispanic, 1.5% other. | All had died within the period of the study.  With dementia = 187.  Without dementia = 1059.  Age not reported. | United States.  Home-based and rest-home care.  Duration of caregiving not reported. | Dementia caregivers had 40.5% higher odds of experiencing increased frailty by the time the death was reported and 90% higher odds in the following wave compared with non-dementia caregivers.  Negative health consequences of caring for a spouse with dementia persist following the cessation of caregiving duties. | A=High  B=High  C=Medium  D= High  Feminist appraisal = low, no discussion of gender. |
| Elizabeth L. Sampson, Rebecca Lodwick, Greta Rait et al. (2016) Living With an Older Person Dying From Cancer, Lung Disease, or Dementia: Health Outcomes From a General Practice Cohort Study, *Journal of Pain and Symptom Management*, 51(5), 839-848. | Undertake a retrospective national cohort study to describe the demographic characteristics, health outcomes, and primary care service use of cohabitees of people dying with cancer (lung or coloretical), dementia, or COPD during the year before and after bereavement. | Retrospective quantitative study using UK primary care database. Analysis using Poisson regression for morbidities. Mulitvariate Cox proportional hazards ratios for mortality. | 13,693 co-habitees of people identified at end-of-life.  Total mean age of carers = 78.  Mean age of carers of someone with: dementia = 82; cancer = 75; COPD = 77.  Gender= two-thirds women.  Ethnicity not reported. | All had died of dementia, cancer or COPD.  Age not reported. | United Kingdom.  Home-based care.  Duration of caregiving not reported. | No significant variation in health outcomes between carers of people with cancer, COPD and dementia.  All experienced significant increase in GP visits and the prescription of anti-depressants.  No mortality differences between the three groups.  Carers significantly under-identified in primary care records. | A=High  B=High  C=High  D= High  Feminist analysis= low, overwhelmingly female sample, no consideration of gender theory. |
| Mary Turner, Claire King, Christine Milligan et al. (2016), Caring for a dying spouse at the end of life: ‘It's one of the things you volunteer for when you get married’: a qualitative study of the oldest carers' experiences, *Age and Ageing*, 45 (3), 421–426. | To explore the experiences of the ‘oldest carers’ in caring for a dying spouse at home. | Cross-sectional qualitative design from a larger qualitative study. Framework and thematic analysis. | 17 spouses who cared for their dying spouse at home.  Age range 80-90.  Gender = 9 women, 8 men.  Ethnicity = White British = 15, White European = 1, White American = 1. | Range of end-of-life conditions: Cancer = 9; dementia = 3; Parkinson’s = 2; Old age = 2; Heart failure = 1; Renal failure = 1.  Mean age = 85.6. | United Kingdom.  Home-based care.  Duration of caregiving: mean = 29 months, median = 11 months. | While spouses wanted to care out of a sense of duty they often were inhibited by their own physical health to do so. Many still providing 24/7 care. Most viewed their role positively. | A= High  B= High  C= Medium  D= High  Feminist analysis= low, no gender analysis. |
| Gwen McGhan, Susan J. Leob, Brenda Baney et al. (2013). End-of-Life Caregiving: Challenges Faced by Older Adult Women. *Journal of Gerontological Nursing*, 39(6), 45-54. | To explore the challenges faced by older adult spousal caregiving providing end-of-life across different life-limiting illness trajectories in distinctive care delivery models. | Qualitative ethnography, instrumental case study. Categorical aggregation analysis. | 3 women caring for a spouse.  Mean age = 77.  Ethnicity not reported. | Partners had ALS, heart failure, lung cancer respectively.  Age not reported. | United States.  Home-based care.  Duration of caregiving not reported.  Mean length of marriage 37 years. | Older adult caregivers had their own age-related health issues that increased the difficulty of providing care. increased the demands of caregiving role and in turn impacted their ability to provide care. The type of care delivery model can improve carer burden. Need to recognise carers as co-recipients of care. | A=High  B= Medium  C=Medium  D= Medium  Feminist analysis= low, explicit focus on women but no focus on gender. |
| Minna Maria Poysti, Marja-Liisa Laakkonen, Timo Strandberg et al. (2012) Gender Differences in Dementia Spousal Caregiving  *International Journal of Alzheimer’s Disease*: 1-5. | Comparison of the characteristics and burden of male and female spousal caregiving of patients with dementia. | Baseline combined data from two intervention trails (around care coordination and exercise respectively). Quantitative Zarit burden scale used for burden and geriatric depression scale used for depression. Logistic regression analysis used. | 335 dyads of wife-husband married couples.  Mean age of carers =78. Mean age of male carers = 77 (SD= 6.2).  Mean age of female carers = 78.5 (SD=5.6).  Gender of carers = 128 men, 207 women.  Ethnicity not reported. | Medium- severe dementia.  Age not reported.  Gender = 128 women, 207 men. | Finland.  Home-based care.  Duration of caregiving not reported. | Male carers have higher rates of comorbidities and cared for partners with more severe dementia than female carers.  Male carers experienced less  burden including depressive symptoms than women carers.  Authors suggest men’s higher sense of personal coherence and lower education levels help explain findings. | A=Low  B= Low  C=Medium  D= Low  Feminist appraisal= moderate  Considers the gendered context of care (albeit without appeal to gender theory). However, assumptions that women are naturally more included to care are reified. |
| Sara Sanders and James Power (2009) Roles, responsibilities, and relationships among older husbands caring for wives with progressive dementia and other chronic conditions *Health & Social Work*, 34, 1: 41-51. | What are the changes in the roles, responsibilities, and relationships that husbands experience as they provide care for their chronically ill wives? | Serial, in-depth qualitative interviews. Phenomenological inductive analysis. | 17 husbands of  Mean age = 77 (SD= 9.5).  Ethnicity = all white. | Wives with moderate to severe dementia and chronic health conditions.  Age not reported. | United States.  Home and rest-home based care.  Duration of caregiving not reported.  Mean length of marriage = 47 years. | Older husbands are intimately involved in the care of their wives, providing personal care and emotional support. They actively try to maintain normalcy within their married lives. The health of their wife heavily impacts nature of caregiving.  Husbands grieved for the impending loss of their partner. | A=High  B=High  C= Medium  D= High  Feminist appraisal  = moderate  Engages with stereotypes of male carers in order to nuance them. Little engagement with gender theory. |
| Helen K. Black, Abby J. Schwartz, Christa J. Caruso et al. (2008), How Personal Control Mediates Suffering: Elderly Husbands’ Narratives of Caregiving, *The Journal of Men’s Studies*, 16(2), 177-192. | To explore oldest-old men’s experiences of suffering in late life. | Qualitative ethnography involving 3 interviews. General qualitative analysis to identify themes. | 4 men caregiving for their  Mean age 84.5.  Ethnicity: African-American = 1; Euro-American = 3. | Wives with moderate to severe dementia.  Age not reported. | United States.  Home-based care.  Mean duration of caregiving = 6 years. | Husbands found ways to cope, with varying degrees of success with caring for their wives.  They tried to maintain their self and marriage identities.  They strove to find or remind themselves of the purpose in their caring. | A=High  B=Low  C=Medium  D= Medium  Feminist analysis= Medium – fit within normative ideas of masculinity e.g. approaching caring like a labor-intensive task and seeking out male carers in their wider sample to explore their particular experience. |
| JoAnn Perry (2002) Wives Giving Care to Husbands with Alzheimer’s Disease: A Process of Interpretive Caring. *Research in Nursing and Health*, 25: 307-316. | To examine the process of becoming and of being a caregiver from the perspective of wives who care for husbands with dementia. | Grounded theory based on symbolic interactionism and using in-depth interviews. Strauss and Corbin’s interpretative analysis employed. | 20 wives.  Mean age = 76.3 (SD 5.61).  Ethnicity of carers: African- American = 3; Euro-American = 17. | Husbands with  middle and advanced stage dementia.  Mean age =78 (SD 6.34). | Washington, United States and British Columbia, Canada.  Home-based care.  Duration of caregiving not reported.  Mean length of marriage = 45 years. | ‘Interpretative caring’ theory emphasising the process of wives’ recognition of husband’s illness.  Wives discussed how they devised strategies to mitigate changes to their daily routine as a result of their husbands’ illness. Emphasised importance of maintaining partners dignity. | A=High  B=Medium  C=High  D= High  Feminist analysis= low, despite talking about wives no mention of the significance of their gender. |
| Richard Russell (2001) In sickness and in health: A qualitative study of elderly men who are for wives with dementia *Journal of Aging Studies* 15: 351-367. | To explore the richness and diversity of experiences encountered by elderly men caregivers, as well as the variety of subjective meanings ascribed to their caregiving experiences. | Qualitative research, inductive analysis. | 14 husbands.  Age range = 68-90 years.  Ethnicity: African-American = 1; Eastern- European = 2; Euro-American = 11. | Wives with dementia (but also inclusive of stroke or brain injury).  Age not reported. | New York, USA.  Home-based care.  Duration of caregiving not reported. | Men are capable, nurturing and innovative carers.  They struggled with aspects of feeling aspects  of their care were invisible.  They drew on formal supports when they needed help. | A= Medium  B= Medium  C= High  D= Medium  Feminist analysis – High. Situates caregiving within changing place of gender studies, deconstructs gender and sex, considers the role of the male interviewer when speaking with men, seeks nuances in men’s answers. |
| George Siriopoulos, Yvonne Brown, Karen Wright (1999) Caregivers of wives diagnosed with Alzheimer’s disease: Husband’s perspectives. *American Journal of Alzheimer’s Disease* 14(2): 79-87. | To look into the experiences and needs of husbands caring for wives with Alzheimer’s disease. | Qualitative phenomenological study. Giorgi’s analysis. | 8 husbands caring for their  Age range of carers = 64-92.  Ethnicity = All white. | Wives with moderate to severe Alzheimer’s disease.  Age range = 68-90. | Alberta, Canada.  Home and rest-home based care.  Duration of caregiving range from 1-10 years. | The commitment and love men felt to their wives underpinned their caregiving.    Caregiving was experienced as a series of losses, some of which were Alzheimer’s specific. | A=High  B= Low  C=High  D= Medium  Feminist analysis – medium, engages with social reproductive theory and Tronto’s idea that care roles are not naturally engrained but doesn’t push analysis further. |
| Helen Edwards & Patricia Noller (1998) Factors Influencing Caregiver- Care Receiver Communication and Its Impact on the Well-Being Older Care Receivers, *Health Communication* 10,4: 317-341. | To examine communication between frail older people and their caregiving spouses, and its relation to well-being in older care receivers. | Quantitative structured survey administered in person and video-taped observation of communication. urvey method with self-reported health and communication tone ratings observations.  Bartlett’s test of sphericity, Kaiser-Meger-Olkin and multiple linear regressions. No ethics recorded. | 53 spousal dyads.  27 caring wives, 26 caring husbands.  Mean age = 74.5 (SD 7.14).  Ethnicity = Majority born in Australia, with a few from United Kingdom and Europe. | Cared-for a range of frailty including cancer, respiratory disease and arthritis. Dementia excluded.  Mean age = 78 (SD 7.35). | Australia.  Home-based care, involved with community care agencies.  Duration of caregiving = 9 years (SD 2.68) | Caregiving wives used more overprotective communication than caregiving husbands.  Carers with low life satisfaction more likely to use patronising communication. | A=High  B= Medium  C=Medium  D= Medium  Feminist analysis= Low, despite flagging discrepancies in expected gendered norms in relation to how women communicated (using language to take control). |
| Sharon L. Tennstedt, Sybil Crawford, and John B. McKinlay (1993) Determining the Pattern of Community Care: Is Coresidence More Important Than Caregiver Relationship? *Journal of Gerontology*, 48(2): 574-583. | To determine whether co-residence is more important than caregiver relationship when explaining amount of care received by frail elderly. | Quantitative, longitudinal telephone survey, with first follow-up (4 yrs after baseline).  Multiple logistic regressions and stepwise modelling procedures. No ethics recorded. | 445 frail elders and their primary carers.  Mean age of carers = 79.  Ethnicity no reported.  Gender of spouses living with partner = 60% women, 40% men. | 55.4% of people living with carer and 34.5% living without had severe frailty.  Median age range of care recipients = 80-84. | Massachusetts, USA.  Home-based care.  Duration of caregiving not reported. | Receipt of informal care is more likely for elders co-residing with a caregiver, regardless of who that person is.  Co-resident caregivers used formal services significantly less. | A= High  B= High  C= Medium  D= High  Feminist analysis- Despite highlighting a number of gendered disparities reported across caregivers and receivers no further theorisation. Makes an argument for equity of services so has broad social justice commitment. |
